# Improvement of Lipid Profile after One-Anastomosis Gastric Bypass Compared to Sleeve Gastrectomy

**DOI:** 10.3390/nu13082770

**Published:** 2021-08-12

**Authors:** Silvia Bettini, Gianni Segato, Luca Prevedello, Roberto Fabris, Chiara Dal Prà, Eva Zabeo, Chiara Compagnin, Fabio De Luca, Cristiano Finco, Mirto Foletto, Roberto Vettor, Luca Busetto, on behalf of the Veneto Obesity Network

**Affiliations:** 1 Department of Medicine, University of Padova, 35128 Padova, Italy; chiara.compagnin@unipd.it (C.C.); fabio.deluca.1@studenti.unipd.it (F.D.L.); roberto.vettor@unipd.it (R.V.); luca.busetto@unipd.it (L.B.); 2 Center for the Study and the Integrated Treatment of Obesity, Padova University Hospital, 35128 Padova, Italy; luca.preve@libero.it (L.P.); roberto.fabris@aopd.veneto.it (R.F.); chiara.dalpra@aopd.veneto.it (C.D.P.); eva.zabeo@aopd.veneto.it (E.Z.); mirto.foletto@aopd.veneto.it (M.F.); 3 Department of General Surgery, “San Bortolo” Hospital, 36100 Vicenza, Italy; gsegat@tin.it; 4 General Surgery, “Cazzavillan” Hospital, Arzignano, 36071 Vicenza, Italy; cristiano.finco@aulss8.veneto.it

**Keywords:** lipid metabolism, one-anastomosis gastric bypass one, sleeve gastrectomy, LDL, HDL, triglycerides, lipoprotein, obesity, dyslipidemia

## Abstract

Fewer studies compared the improvement of plasma lipid levels after different types of surgery, in particular compared to one-anastomosis gastric bypass (OAGB). The aim of our study was to investigate how laparoscopic sleeve gastrectomy (LSG) and OAGB impact on weight loss and lipid profile 18 months after surgery, in patients with severe obesity. Forty-six patients treated with OAGB were matched to eighty-eight patients submitted to LSG. Weight loss after OAGB (33.2%) was more evident than after LSG (29.6%) (*p* = 0.024). The difference in the prevalence of dyslipidemia showed a statistically significant reduction only after OAGB (61% versus 22%, *p* < 0.001). After adjustment for delta body mass index (BMI), age and sex, we demonstrated a statistically significant decrease of the differences between the changes before and after (delta Δ) the two surgery procedures: Δ total cholesterol values (*p* < 0.001), Δ low density lipoprotein-cholesterol values (*p* < 0.001) and Δ triglycerides values (*p* = 0.007). Patients with severe obesity undergoing to OAGB presented a better improvement of lipid plasma values than LSG patients. The reduction of lipid plasma levels was independent of the significant decrease of BMI after surgery, of age and of sex.

## 1. Introduction

Several studies have shown the efficacy of bariatric surgery, not only in terms of weight loss, but also remission from type 2 diabetes mellitus (T2DM) and decrease of cardiovascular risk [1].

The most commonly performed procedure is laparoscopic sleeve gastrectomy (LSG), followed by Roux-en-Y gastric bypass (RYGB) [2]. One anastomosis gastric bypass (OAGB), representing approximately 4.8% of bariatric surgeries worldwide, is a new modified gastric bypass that consists of a single gastrojejunal anastomosis between a long gastric pouch and a jejunal omega loop [3,4,5]. The efficacy and safety of OAGB versus RYGB for obesity has been demonstrated in the YOMEGA study [4]. The biliopancreatic limb length has proved to be essential to weight loss, comorbidity resolution and nutritional deficiencies and a 150 cm limb length is adequate for assuring a good equilibrium between weight loss and nutritional complications [6].

A systematic review and meta-analysis to examine the effectiveness and risks of bariatric surgery showed that only few studies investigated dyslipidemia outcome; however, more than two thirds out of the patients included in these studies showed remission of dyslipidemia after surgery [7]. Moreover, several studies compared outcomes of different surgical procedures in terms of weight loss, complications, quality of life and remission of comorbidities, in particular T2DM, hypertension and obstructive sleep apnea [8,9,10,11,12]. Conversely, fewer studies compared the improvement of plasma lipid levels after different types of surgery [13], in particular compared to OAGB [9,14].

The aim of our study was to compare weight loss and the impact on improvement of lipid profile 18 months after LSG and OAGB in patients with obesity.

## 2. Materials and Methods

### 2.1. Patients and Settings

This study was conducted in two bariatric centers belonging to the Veneto Obesity Network: the Center for the Study and the Integrated Treatment of Obesity (CeSTIO) at the Padova University Hospital, Padova and the General Surgery at the Arzignano Hospital, Arzignano, Vicenza. The Obesity Veneto Network (Rete Veneta Obesità) is the clinical network offering medical and surgical treatment of obesity in the Veneto Region, Italy. The network consists of a Coordinating Center in Padua, two Hub Centers (University Hospitals located in Padova and Verona), which contain all the specializations and Spoke Centers (such as Arzignano) distributed throughout the region.

Fifty-nine consecutive patients treated with OAGB at the Arzignano Hospital were included in the period between 2011–2018. Other one hundred and one consecutive patients treated with LSG at the CeSTIO were included in the same period. These two subgroups of patients were matched according to age, sex and Body Mass Index (BMI) values and we, finally, selected 46 patients in the group OAGB and 88 patients in the group LSG. All patients underwent a multi-disciplinary evaluation according to a standardized clinical protocol and were consequently assigned to surgical or medical treatment according to European criteria [15]. The assignment to LSG or OAGB was in accordance with surgical preference at the respective center.

Patients were eligible for inclusion if their BMI was 40 kg/m^2^ or higher, or 35 kg/m^2^ or higher with the presence of at least one comorbidity (T2DM, hypertension, obstructive sleep apnea, dyslipidemia, or arthritis) and were aged 18–65 years. Bariatric procedures were done laparoscopically and performed by the same surgeons by using the same surgical technique. LSG involved stomach longitudinal resection starting 4–5 cm from the pylorus with the preservation of the gastric antrum. The procedure was calibrated upon a 34-Fr gastric bougie [16]. OAGB consisted of a long gastric tube beginning at the incisura angularis and calibrated with a 37 French bougie. A single gastrojejunal anastomosis required a linear stapler with a biliopancreatic limb of 150 cm [3].

All participants were followed-up for almost 2 years by the respective centers and for the present study we considered a follow-up of 18 ± 6 months. All patients were recommended to follow a balanced hypocaloric diet (25–30% fat, 50–55% carbohydrates, 20% protein) and physical activity prescriptions (150 min per week of moderate intensity physical activity). Patients received the same dietary counseling, psychotherapy and physical training, according to an internal therapeutic care plan, of the Veneto Obesity Network (DRG *n*° 26, 9 March 2017). Baseline evaluation was performed within 6 months before surgery. We classified our population according to patients’ glycaemic profile in normal glycaemia, affected by prediabetes (pre-DM) and T2DM [17]. Diagnosis of arterial hypertension and dyslipidemia was based on recent guidelines [18,19].

All patients provided written informed consent in accordance with the Declaration of Helsinki. The protocol was approved by the ‘Padua Ethical Committee for Clinical Research’ (2892P, 10 June 2013).

### 2.2. Anthropometric Measurements

Each patient’s BMI was calculated as the ratio of weight (kg) to height squared (m^2^), with a precision of 0.1 kg and 0.01 m. All anthropometric measurements were assessed with patients wearing light clothes without shoes. Waist circumference was assessed using a tape measure and we were not able to record waist circumference measures after OAGB.

### 2.3. Biochemical Measurements

All biochemical measurements were performed after 8 h fasting, with standard diagnostic kit according to the WHO First International Reference Standard: fasting plasma glucose (FPG) (Glucose HK Gen.3, Roche Diagnostic, Indianapolis, IN, USA), insulin (IMMULITE 2000 Immunoassay, Siemens Healthcare GmbH, Erlangen, Germany), glycated hemoglobin A1c (Hb1Ac) (HPLC) (only in patients with T2DM). Alanine aminotransferase (ALT), aspartate aminotransferase (AST) and gamma glutamiltrasferase (GGT) were assayed by enzymatic method with the addition of pyridoxal-5-phosphate in compliance with IFCC reference methods [20]. Serum lipids (total cholesterol (TC), high density lipoprotein-cholesterol (HDL), triglycerides (TG)) were measured by spectrophotometer (Cobas 8000, Roche Diagnostic, Indianapolis, IN, USA). Low density lipoprotein-cholesterol (LDL) was calculated according to Friedewald [21]. Non-HDL-cholesterol (NHDLC) was calculated as the difference between TC and HDL. The homeostasis model assessment (HOMA) was used as index of insulin-resistance, as follows: [fasting serum insulin (μU/mL) x fasting plasma glucose (mmol/L)]/22.5 [22]. In patients affected by T2DM, if insulin treated, fasting insulin was not measured and HOMA was not calculated. In OAGB group we were not able to collect insulin values in the follow-up and thus, HOMA was not calculated. The fatty liver index (FLI) was calculated as: FLI = (e ^0.953 * loge (triglycerides) + 0.139 * BMI + 0.718 * loge (ggt) + 0.053*waist circumference − 15.745^)/(1 + e ^0.953 * loge (triglycerides) + 0.139 * BMI + 0.718 * loge (ggt) + 0.053 * waist circumference − 15.745^) * 100 [23].

### 2.4. Statistical Analysis

Statistical analyses were computed using the SigmaPlot v.14 (Systat Software, Adalta, Arezzo, Italy). All variables were tested by normal Test and Equal Variance Test (Shapiro–Wilk test and Brown–Forsythe, respectively). For the comparison of continuous variables t-test or Mann–Whitney Rank Sum Test were used according to their distribution. For the comparison of categorial variables Chi-square test were utilized. Analysis of variables before and after bariatric surgery were tested by Paired Student’s *t*-test or Wilcoxon Signed Rank Test (continuous variables), according their distribution and by Chi-square test (categorial variables). A multiple regression model was used to adjust the difference of parameters of the lipid profile (TC, HDL, CNHDL, LDL, TG) before and after surgery for the difference of BMI, sex and age. In order of this, we considered each parameter of lipid profile of both the surgeries (i.e., we performed 10 multiple linear regressions) as a dependent variable and delta BMI, age and sex as independent variables. When one or more of these independent variables entered in the final equation (i.e., the *p* was <0.05) we used them to correct the value of each lipid parameter. Variant inflation factor was considered as measure of multicollinearity. In all analyses, the *p* values were two-sided and a *p* value lower than 0.05 was considered statistically significant.

## 3. Results

Basal characteristics of patients underwent to LSG (*n* = 88) and OAGB (*n* = 46) were shown in Table 1.

The mean age of the patients at baseline was 47.9 ± 10.5 years and 46.5 ± 8.8 years for LSG and OAGB, respectively (*p* = 0.473). The median pre-operative BMI was 46 (interquartile range, IQR, 41.6–51.7) and 44.9 (IQR 41.2–48.1), respectively, with no significant difference between the two groups (*p* = 0.159). Before surgery, 52% of the patients of the LSG group and 61% of the patients of the OAGB group had dyslipidemia (*p* = 0.443). The metabolic impairment, meaning the presence of prediabetes and diabetes and the presence of hypertension were not different among the two populations (*p* = 0.479, *p* = 0.210, respectively). Indeed, the use of anti-dyslipidemic drugs were similar between groups. The only parameter being different among the two subgroups was the TG values, with OAGB group presenting the higher ones (*p* = 0.007) (Table 1).

Table 2 and Table 3 showed the changes in the anthropometric, biochemical parameters and comorbidities 18 months after the two procedures. Weight loss after LSG was 29.6% and it was accompanied by a statistically significant reduction of waist circumference (19.8%, *p* < 0.001), FPG (14.8%, *p* < 0.001), ALT (26.1%, *p* < 0.001), AST (22.7%, *p* < 0.001), HDL (20.4%, *p* < 0.001), NHDLC (11.4%, *p* < 0.001), LDL (7.8%, *p* < 0.001) and TG (24.2%, *p* < 0.001). After OAGB, weight loss was more evident (33.2%) with a deeper decrease of lipid profile levels: CT (12.6%, *p* < 0.001), HDL (16.6%, *p* < 0.001), NHDLC (20.6%, *p* < 0.001), LDL (16.8%, *p* < 0.001) and TG (32.6%, *p* < 0.001). After OAGB, we observed a statistical significantly decrease in FPG levels (12.7%, *p* < 0.001), while transaminase did not reduce. After LSG we noticed a relevant improvement of metabolic profile and a decrease in prevalence of prediabetes and diabetes (*p* < 0.001) and a statistically significant decrease in prevalence of hypertension (*p* < 0.001). Consensually, after OAGB, we described a similar decrease in prevalence of prediabetes and diabetes (*p* < 0.01), whereas the prevalence of hypertension remained unchanged. Finally, to corroborate the improvement of transaminases only after LSG, we calculated FLI before (94.89 ± 8.77) and after LSG (55.15 ± 29.34), resulting in a statistically significant decrease (*p* < 0.001).

**Figure 1:**
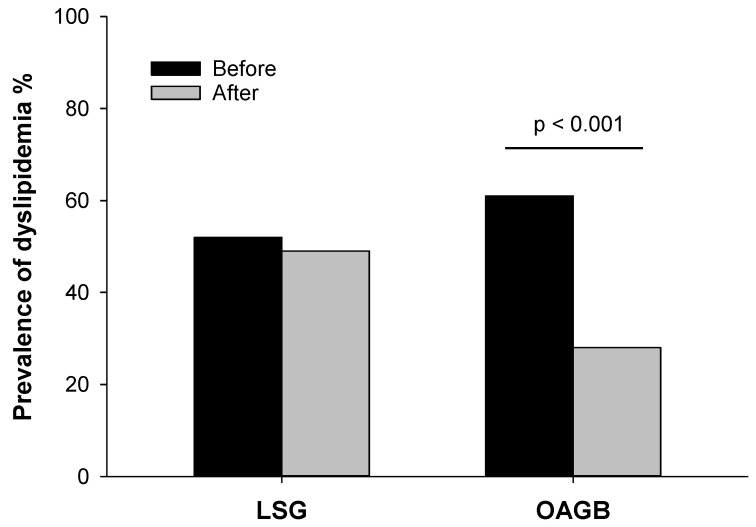
Prevalence (percentage) of dyslipidemia before (black bars) and after (grey bars) laparoscopic sleeve gastrectomy (LSG) (*n* = 88) and one anastomosis gastric bypass (OAGB) (*n* = 46). Statistical analysis was performed by Chi-square test.

Figure 1 represented the difference in the prevalence of dyslipidemia after LSG (before surgery 52%, after surgery 49%, *p* = 0.763) and OAGB, showing a statistically significant slowing only after OAGB (before surgery 61%, after surgery 22%, *p* < 0.001).

**Figure 2:**
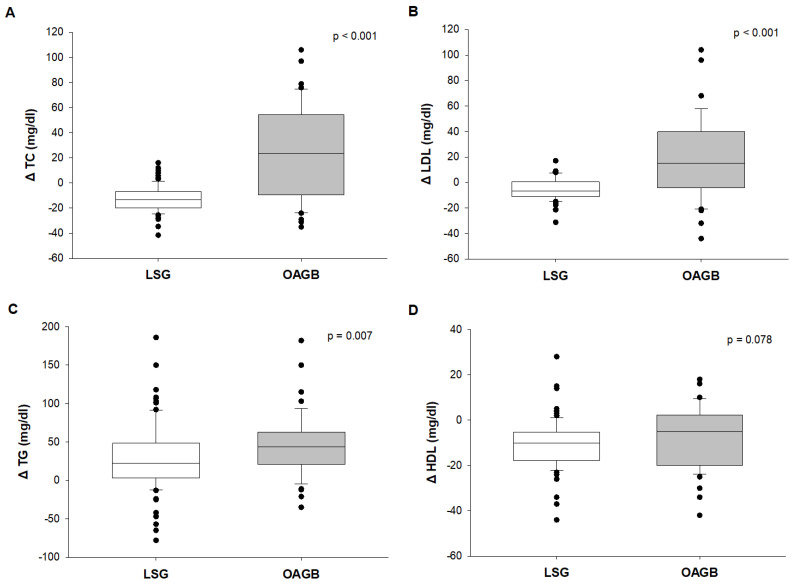
Difference between the changes (delta Δ) of Total Cholesterol (TC) (**A**), triglycerides (TG) (**B**), Low Density Lipoprotein-cholesterol (LDL) (**C**) and High Density Lipoprotein -cholesterol (HDL) (**D**) before and after Laparoscopic Sleeve Gastrectomy (LSG) and One Anastomosis Gastric Bypass (OAGB). Statistical analysis was performed by Mann-Whitney Rank Sum Test for the comparison between delta; multiple regression model for the adjustment for Δ BMI, sex and age.

Table 4 highlighted the differences between the changes before and after (delta, Δ) the two surgery procedures. The difference in weight loss was greater after OAGB than LSG (Δ weight: 42.0 kg versus 37.6 kg, *p* = 0.024) with a corresponding statistically significant decrease of Δ BMI (15.5 kg/m^2^ versus 13.7 kg/m^2^, *p* = 0.032). The difference in each plasma lipid levels was expressed both without and after adjustment for delta BMI, age and sex. We demonstrated a statistically significant decrease of Δ TC values (*p* < 0.001, Table 4 and Figure 2A), Δ NHDLC values (*p* < 0.001, Table 4), Δ LDL values (*p* < 0.001, Table 4 and Figure 2B) and Δ TG values (*p* = 0.007, Table 4 and Figure 2C), after adjustment for delta BMI, age and sex. On the other hand, Δ HDL adjusted values showed an increase, not reaching a statistically significant evidence (*p* = 0.078, Table 4 and Figure 2D).

## 4. Discussion

While the efficacy and safety of OAGB have been validated [3,4,5] and its ability to control dyslipidemia has now been demonstrated [9,14,24], this approach is included in the ongoing debate on which procedure is the most effective in such patients. A moderate amount of studies focused on remission of T2DM, comparing OAGB with other surgical procedures [8,9,11,25,26,27]. Thus, this study attempts to compare LSG and OAGB in term of improvement of lipid profile in patients with severe obesity. We demonstrated that plasma lipid levels decrease more after OAGB compared to LSG, in patients with similar preoperative characteristics, independently of age, sex and the reduction of BMI.

It was already known that bariatric surgery induces remission from comorbidities and, nevertheless less investigated, also from dyslipidemia. A systematic review and meta-analysis to examine the effectiveness and risks of bariatric surgery showed that randomized control trials displayed 76% remission of dyslipidemia after surgery and in observational studies, the remission rate was 68% [7]. In a prospective randomized double-blind trial, silastic ring RYGB resulted in significantly lower total cholesterol and LDL cholesterol levels than LSG [12]. These results are consistent with another recent meta-analysis [28] that demonstrated TC and LDL levels are reduced within 1 month, while HDL levels increase after 12 months and TG levels decrease significantly after 3 months. This study showed that the most important improvement in plasma lipids following RYGB surgery occurs in the first follow-up, when patients did not yet achieve a substantial weight loss. It was demonstrated that in Indian patients with obesity both LSG and RYGB may be considered effective for dyslipidemia improvement [13].

Fewer studies compared different outcome between LSG and OAGB. A study aiming to determine the efficacies of OAGB and LSG on diabetic control showed that OAGB group had a significantly lower blood lipid levels than the LSG group at 12 months after surgery [9]. Five-year outcomes of LSG and OAGB in terms of weight loss and regain, complications and resolution of comorbidities described a dyslipidemia remission of 72% after LSG and 90% after OAGB [14]. Consensually, in a 7-year-follow-up study remission of dyslipidemia was also higher in the OAGB group (74%) compared to LSG group (52%) [29].

In our study we compared comorbidities outcome between LSG and OAGB, focusing on dyslipidemia. Both after LSG and OAGB we noticed a relevant improvement of metabolic profile and a decrease in prevalence of prediabetes and diabetes. Consensually, also FPG and Hb1Ac levels decreased significantly. Regarding hypertension, after LSG we showed a statistically significant decrease in its prevalence. On the other hand, after OAGB, the prevalence of hypertension remained unchanged. The transaminases values ameliorated only after LSG; considering the lack of liver ultrasound after surgery in this study, we consider FLI a useful score to demonstrate the improvement of liver steatosis after LSG [23].

Furthermore, we demonstrated that prevalence of dyslipidemia statistically significant decreased only in the OAGB group, even if the lipid profile values decreased in both groups. Comparing the difference of each lipid profile value before and after surgery, we evidenced a decrease of TC, LDL, NHDLC and TG levels in the OAGB group greater than the LSG group. Furthermore, HDL levels increased more after OAGB compared to LSG even if the difference did not reach the statistical significance. On the contrary to our results, other studies, comparing the effects on lipid profile 12 months after LSG and OAGB, showed the same effectiveness of the two procedures for dyslipidemia improvement [10,25]. On the other hand, two studies confirmed our findings [9,14] and, to our knowledge, we firstly proved that these differences were independent of age, sex and difference in weight loss level. In other words, our study showed that the improvement of lipid profile may be a direct consequence of the surgery rather than weight loss, per se.

It was demonstrated that OAGB induced a wider weight loss than LSG in both short and long follow-up [10,11,25,26]. In our study, substantial weight loss was achieved for patients in both subgroups, with OAGB patients obtaining the greater slowing. Nevertheless, we highlighted that the improvements of lipid plasma values in OAGB patients was independent of the reached BMI.

OAGB surgery produces a long gastric tube with faster passage of freshly chewed, but undigested food, through a tract of intestine that was not used to receiving it in this form and consistency. This modification results in a reduction of emulsification and lipid absorption in the ileum. It could be suggested that a reduction in cholesterol absorption, already demonstrated after RYGB [30], may be present also after OAGB. Indeed, considering that lipids do not go through duodenum anymore, a reduction in bile acids secretion may be present after OAGB. Another factor influencing the reduction of plasma lipids after OAGB is the reduction in food intake, which can be explained by loss of appetite, early satiety and changes in gut hormones. Thus, not only restriction and malabsorption but also other mechanisms are being suggested to determine the outcome of bariatric surgery in ameliorating obesity-related comorbidities. These mechanisms are related to weight-independent factors such as gut hormones, the nervous system, bile acids and the gut microbiota, as well as the interactions between them and they have been studied in particular in term of remission from T2DM [31]. Although the resolution of dyslipidemia has been less investigated, Carbajo et al. [32] found that, despite of maximum weight loss, observed during the first 6-month follow-up, LDL levels progressively decreased within 2 years after surgery. This study demonstrated that LDL levels at the end of follow-up are independent of weight loss.

Our study has some limitations. Because of our selection criteria, we casually obtained an OAGB group with baseline TG plasma values being statistically significant difference from LSG group. However, this did not impact on the results of our study. Another limitation is the lack of a long-term follow-up that demonstrate the persistence of improvement of lipid plasma levels after bariatric surgery. Finally, we were not able to collect data on cardiovascular outcome, beyond data on prevalence of hypertension.

In conclusion, we demonstrated that patients with severe obesity undergoing to OAGB presented a better improvement of lipid plasma values than LSG patients. This fact was accompanied by a substantial remission by dyslipidemia only in the OAGB group. The reduction of lipid plasma levels was independent of the significant decrease of BMI after surgery, of age and of sex. Since OAGB resulted more effective than LSG in the management of dyslipidemia, we suggest that a surgical procedure that includes a bypass component (such as OAGB) could be tailored to patients with severe obesity and a worst control of plasma lipids.

## Figures and Tables

**Table 1:** Basal characteristics of patients underwent to laparoscopic sleeve gastrectomy (LSG) and one anastomosis gastric bypass (OAGB).

	LSG (*n* = 88)	OAGB (*n* = 46)	*p*
**Sex (M/F)**	20/68	4/42	0.076
**Age (years)**	47.9 ± 10.5	46.5 ± 8.8	0.473
**Weight (kg)**	127 (108.6–144.8)	123.5 (112.3–137)	0.598
**BMI (kg/m^2^)**	46 (41.6–51.7)	44.9 (41.2–48.1)	0.159
**Waist (cm)**	127 (120.8–144)	125.5 (116–132)	0.094
**TC (mg/dL)**	186 (166–205)	187 (167–207)	0.752
**HDL (mg/dL)**	49 (39–56)	47 (37–59)	0.699
**NHDLC (mg/dL)**	137 (115–156)	145 (115–161)	0.689
**LDL (mg/dL)**	115 (97–135)	118 (91–130)	0.591
**TG (mg/dL)**	95 (70–134)	129 (92–165)	**0.007**
**HOMA-IR**	3.6 (2–6)	4.7 (2.9–9.5)	0.092
**FPG (mmol/L)**	5.4 (5–6.2)	5.5 (4.9–6.3)	0.528
**Insulin (mU/L)**	13.5 (7.3–23.3)	16.5 (10.3–33.6)	0.163
**Hb1Ac (%)**	6.9 (6.2–7.6)	6.1 (5.8–8.2)	0.696
**ALT (U/L)**	23 (17–33)	23 (19–35)	0.757
**AST (U/L)**	22 (17–30)	18 (16–24)	0.059
**Metabolic profile (*N, preDM, T2DM*)**	45 (51%)/22 (25%)/21 (24%)	23 (50%)/14 (30%)/9 (20%)	0.479
**Dyslipidemia**	46 (52%)	28 (61%)	0.443
**Hypertension**	42 (48%)	18 (39%)	0.737
**Anti-dyslipidemic drugs** *	18 (20%)	4 (9%)	0.134

M: male, F: female, BMI: Body Mass Index, TC: Total Cholesterol, HDL: High Density Lipoprotein -cholesterol, NHDLC: non HDL-cholesterol, LDL: Low Density Lipoprotein-cholesterol, TG: triglycerides, FPG: Fasting plasma glucose, Hb1Ac: glycated hemoglobin A1c (only in patients with Type 2 Diabetes Mellitus), HOMA-IR: Homeostasis model assessment-insulin resistance, ALT: alanine aminotransferase, AST: aspartate aminotransferase, N: normal glycemia, preDM: prediabetes, T2DM: Type 2 Diabetes Mellitus. * statins and/or ezetimibe. Statistical Analysis: for continuous variables *t*-test or Mann-Whitney Rank Sum Test; for categorial variables Chi-square test. Significant values are represented in bold.

**Table 2:** Anthropometric, biochemical parameters and comorbidities change 18 months after laparoscopic sleeve gastrectomy in 88 patients.

	Before	After	Delta
**Weight (kg)**	127 (108.6–144.8)	92.2 (74.1–105.8) ***	37.6 (24.2–51.1), 29.6%
**BMI (kg/m^2^)**	47.4 ± 8.2	33.7 ± 7 ***	13.7 ± 5.4, 28.9%
**Waist (cm)**	131.1 ± 16.3	105.1 ± 16.7 ***	26 ± 12.3, 19.8%
**TC (mg/dL)**	189 ± 40	182 ± 37	7 ± 32, 3.7%
**HDL (mg/dL)**	49 (39–56)	57 (49–69) ***	−10 ((−18)–(−5)), 20.4%
**NHDLC (mg/dL)**	140 ± 38	123 ± 35 ***	16 ± 30, 11.4%
**LDL (mg/dL)**	115 (97–135)	103 (85–123) ***	9 ((−1)–(24)), 7.8%
**TG (mg/dL)**	95 (70–134)	75 (57–98) ***	23 (3–49), 24.2%
**FPG (mmol/L)**	5.4 (5–6.2)	4.6 (4.4–4.9) ***	0.8 (0.5–1.3), 14.8%
**Hb1Ac (%)**	6.9 (6.2–7.6)	5.9 (5.2–6.3) ***	1 (0.5–2), 19%
**ALT (U/L)**	23 (17–33)	16 (12–20) ***	6 (2–15), 26.1%
**AST (U/L)**	22 (17–30)	16 (14–20) ***	5 (1–12), 22.7%
**Metabolic profile (*N, preDM, T2DM*)**	45 (51%)/22 (25%)/21 (24%)	71 (81%)/13 (15%)/4 (4%) ***	-
**Hypertension**	42 (48%)	25 (28%) ***	-
**Anti-dyslipidemic drugs** *	18 (20%)	7 (8) ***	-

BMI: Body Mass Index, TC: Total Cholesterol, HDL: High Density Lipoprotein-cholesterol, NHDLC: non HDL-cholesterol, LDL: Low Density Lipoprotein-cholesterol, TG: triglycerides, FPG: Fasting plasma glucose, Hb1Ac: glycated hemoglobin A1c (only in patients with Type 2 Diabetes Mellitus), ALT: alanine aminotransferase, AST: aspartate aminotransferase. * statins and/or ezetimibe. Statistical Analysis: for continuous variables Paired Student’s *t*-test Before vs. After or Wilcoxon Signed Rank Test; for categorial variables Chi-square test. *** *p* < 0.001.

**Table 3:** Anthropometric, biochemical parameters and comorbidities change 18 months after One Anastomosis Gastric Bypass in 46 patients.

	Before	After	Delta
**Weight (kg)**	126.5 ± 18.4	83.3 ± 14.2 ***	42 (33–50), 33.2%
**BMI (kg/m^2^)**	45.2 ± 6.1	29.8 ± 4.6 ***	15.5 ± 4, 34.3%
**TC (mg/dL)**	190 ± 33	166 ± 27 ***	24 ± 36, 12.6%
**HDL (mg/dL)**	48 ± 12	55 ± 13 ***	−8 ± 13, 16.6%
**NHDLC (mg/dL)**	141 ± 33	111 ± 25 ***	29 ± 34, 20.6%
**LDL (mg/dL)**	113 ± 32	95 ± 24 ***	19 ± 32, 16.8%
**TG (mg/dL)**	129 (92–164)	84 (62–107) ***	42 (8–59), 32.6%
**FPG (mmol/L)**	5.5 (4.9–6.3)	4.7 (4.4–5) ***	0.7 (0.2–1.8), 12.7%
**Hb1Ac (%)**	6.1 (5.8–8.2)	5.6 (5–6) *	0.5 (0.5–1.9), 8%
**ALT (U/L)**	23 (19–35)	27 (19–33)	−4 ((−10)–(8)), 17.4%
**AST (U/L)**	22 ± 12	25 ± 10	−4 ((−12)–(2)), 18.2%
**Metabolic profile (*N, preDM, T2DM*)**	23 (50%)/14 (30%)/9 (20%)	37 (80%)/7 (15%)/2 (5%) **	-
**Hypertension**	18 (39%)	18 (39%)	-
**Anti-dyslipidemic drugs** *	4 (9%)	1 (2%)	

BMI: Body Mass Index, TC: Total Cholesterol, HDL: High Density Lipoprotein -cholesterol, NHDLC: non HDL-cholesterol, LDL: Low Density Lipoprotein-cholesterol, TG: triglycerides, FPG: Fasting plasma glucose, Hb1Ac: glycated hemoglobin A1c (only in patients with Type 2 Diabetes Mellitus), ALT: alanine aminotransferase, AST: aspartate aminotransferase. * statins and/or ezetimibe. Statistical Analysis: for continuous variables Paired Student’s *t*-test Before vs. After or Wilcoxon Signed Rank Test; for categorial variables Chi-square test. * *p* < 0.05, ** *p* < 0.01, *** *p* < 0.001.

**Table 4:** Difference between the changes (delta Δ), before and after laparoscopic sleeve gastrectomy (LSG) and one anastomosis gastric bypass (OAGB).

	LSG	OAGB	*p*
**Δ Weight (kg)**	37.6 (24.2–51.1)	42 (33–50)	**0.024**
**Δ BMI (kg/m^2^)**	13.7 ± 5.4	15.5 ± 4	**0.032**
**Δ TC (mg/dL)**	7 ± 32	24 ± 36	**0.009**
**Δ TC adj (mg/dL)**	−13 ((−20)–(−7))	24 ((−9)–54)	**<0.001**
**Δ HDL (mg/dL)**	−10 ((−18)–(−5))	−5 ((−20)–2)	0.078
**Δ HDL adj (mg/dL)**	−10 ((−17.8)–(−5.3))	−5 ((−20)–(2.3))	0.078
**Δ NHDLC (mg/dL)**	16 ± 30	29 ± 34	**0.032**
**Δ NHDLC adj (mg/dL)**	−7 ((−15–0)	27 (4–53)	**<0.001**
**Δ LDL (mg/dL)**	9 ((−1)–(24))	15 ((−4)–40)	0.218
**Δ LDL adj (mg/dL)**	−7 ((−11)–1)	15 ((−4)–40)	**<0.001**
**Δ TG (mg/dL)**	23 (3–49)	42 (8–59)	**0.034**
**Δ TG adj (mg/dL)**	22 (3–49)	44 (21–63)	**0.007**

BMI: Body Mass Index, TC: Total Cholesterol, HDL: High Density Lipoprotein -cholesterol, NHDLC: non HDL-cholesterol, LDL: Low Density Lipoprotein-cholesterol, TG: triglycerides, adj: adjustment. Statistical Analysis: Mann-Whitney Rank Sum Test for the comparison between delta; multiple regression model for the adjustment for Δ BMI, sex and age. Significant values are represented in bold.

## Data Availability

Data were extracted from the electronic clinical records and patient confidentiality was protected by assigning an anonymous identification code.
